# Causal effects of sepsis on structural changes in cerebral cortex: A Mendelian randomization investigation

**DOI:** 10.1097/MD.0000000000039404

**Published:** 2024-09-06

**Authors:** Dengfeng Zhou, Weina Wang, Jiaying Gu, Qiaofa Lu

**Affiliations:** a Department of Respiratory and Critical Care Medicine, Wuhan Fourth Hospital, Wuhan, Hubei Province, China.

**Keywords:** brain structures, causality, Mendelian randomization, sepsis

## Abstract

Previous research has shown a strong correlation between sepsis and brain structure. However, whether this relationship represents a causality remains elusive. In this study, we employed Mendelian randomization (MR) to probe the associations of genetically predicted sepsis and sepsis-related death with structural changes in specific brain regions. Genome-wide association study (GWAS) data for sepsis phenotypes (sepsis and sepsis-related death) were obtained from the IEU OpenGWAS. Correspondingly, GWAS data for brain structural traits (volume of the subcortical structure, cortical thickness, and surface area) were derived from the ENIGMA consortium. Inverse variance weighted was mainly utilized to assess the causal effects, while weighted median and MR-Egger regression served as complementary methods. Sensitivity analyses were implemented with Cochran *Q* test, MR-Egger regression, and MR-PRESSO. In addition, a reverse MR analysis was carried out to assess the possibility of reverse causation. We identified that genetic liability to sepsis was normally significantly associated with a reduced surface area of the postcentral gyrus (β = −35.5280, SE = 13.7465, *P* = .0096). The genetic liability to sepsis-related death showed a suggestive positive correlation with the surface area of fusiform gyrus (β = 11.0920, SE = 3.6412, *P* = .0023) and posterior cingulate gyrus (β = 3.6530, SE = 1.6684, *P* = .0286), While it presented a suggestive negative correlation with surface area of the caudal middle frontal gyrus (β = −11.4586, SE = 5.1501, *P* = .0261) and frontal pole (β = −1.0024, SE = 0.4329, *P* = .0206). We also indicated a possible bidirectional causal association between genetic liability to sepsis-related death and the thickness of the transverse temporal gyrus. Sensitivity analyses verified the robustness of the above associations. These findings suggested that genetically determined liability to sepsis might influence the specific brain structure in a causal way, offering new perspectives to investigate the mechanism of sepsis-related neuropsychiatric disorders.

## 1. Introduction

Sepsis is one of the leading causes of death worldwide, posing a serious threat to public health.^[1]^ According to epidemiological surveys, sepsis affected nearly 48.9 million people worldwide in 2017, with 11 million deaths, accounting for 19.7% of global fatalities.^[2]^ Sepsis is an intrinsically complex and heterogeneous disease, characterized by multi-organ dysfunction including the brain.^[3]^ Acute brain dysfunction caused by sepsis, known as sepsis-associated encephalopathy, is a common complication during sepsis with an incidence rate of up to 70%.^[4]^ It is manifested as delirium and coma closely, which is associated with increased mortality rates.^[4]^ More concerning, sepsis is a major risk factor for long-term cognitive disorders. Hospitalization for severe sepsis increases the incidence of moderate to severe cognitive dysfunction by over 10%.^[5,6]^ Also, the incidence of mental disorders such as depression, anxiety, and posttraumatic stress disorder among sepsis survivors is significantly higher than that in the general population.^[7]^ These findings suggested that sepsis severely impacts the quality of daily life for sepsis survivors, imposing a substantial healthcare burden on families and society. Therefore, it is of great significance to fully explore the neuropsychiatric diseases associated with sepsis to reduce the mortality and improve the survival quality of sepsis.

The brain, including the cortex and subcortical structures, is the human’s highest neural center, responsible for various functions such as movement, sensation, and cognition. Brain structural changes are thought to be the basis for functional changes, with specific brain structure patterns always observed in various neuropsychiatric diseases. A growing number of studies have shown a correlation between sepsis and brain structures.^[8–11]^ For example, a study based on brain magnetic resonance imaging (MRI) revealed that atrophy occurred in 60% of sepsis patients with acute brain dysfunction, especially in areas like cerebral and cerebellar white matter, cortex, hippocampus, and amygdala.^[8]^ Reductions in the volume of the caudate, putamen, and thalamus were associated with poor prognosis of sepsis.^[8]^ Furthermore, Gunther et al showed that sepsis survivors experienced general or partial brain atrophy after discharge, with the degree of atrophy in specific brain regions being associated with reduced cognitive function, executive function, and visual attention disorders.^[11]^ However, the causal link between sepsis and brain structures remains uncertain due to traditional observational studies are prone to biases from confounding factors and reverse causation.

Mendelian randomization (MR) analysis is a new epidemiological method utilizing genetic variants as instrumental variables (IVs) for investigating causality between exposures and outcomes.^[12]^ Based on the law of segregation, it is assumed that genetic variants are randomly distributed to offspring before birth, akin to the random assignment in randomized clinical trials, thereby mitigating the bias of confounding factors and reverse causation.^[13]^ So far, MR has been employed to investigate the causal effect of various exposures (such as disease exposures including schizophrenia and other neuropsychiatric disorders, lifestyle behaviors, and circulating biomarkers) on brain structures.^[14–16]^ However, the causal relationship between sepsis and cortical brain structures has not yet been examined. In this study, we employed a MR analysis to explore the causal effects of genetic liability to sepsis and sepsis-related death on cortical and subcortical structures (34 regional cortical thickness and surface area, 7 subcortical volume). Findings from this study may provide new perspectives on the relationship between sepsis and neuropsychiatric disorders.

## 2. Materials and methods

### 2.1. Study design

**Figure 1.**
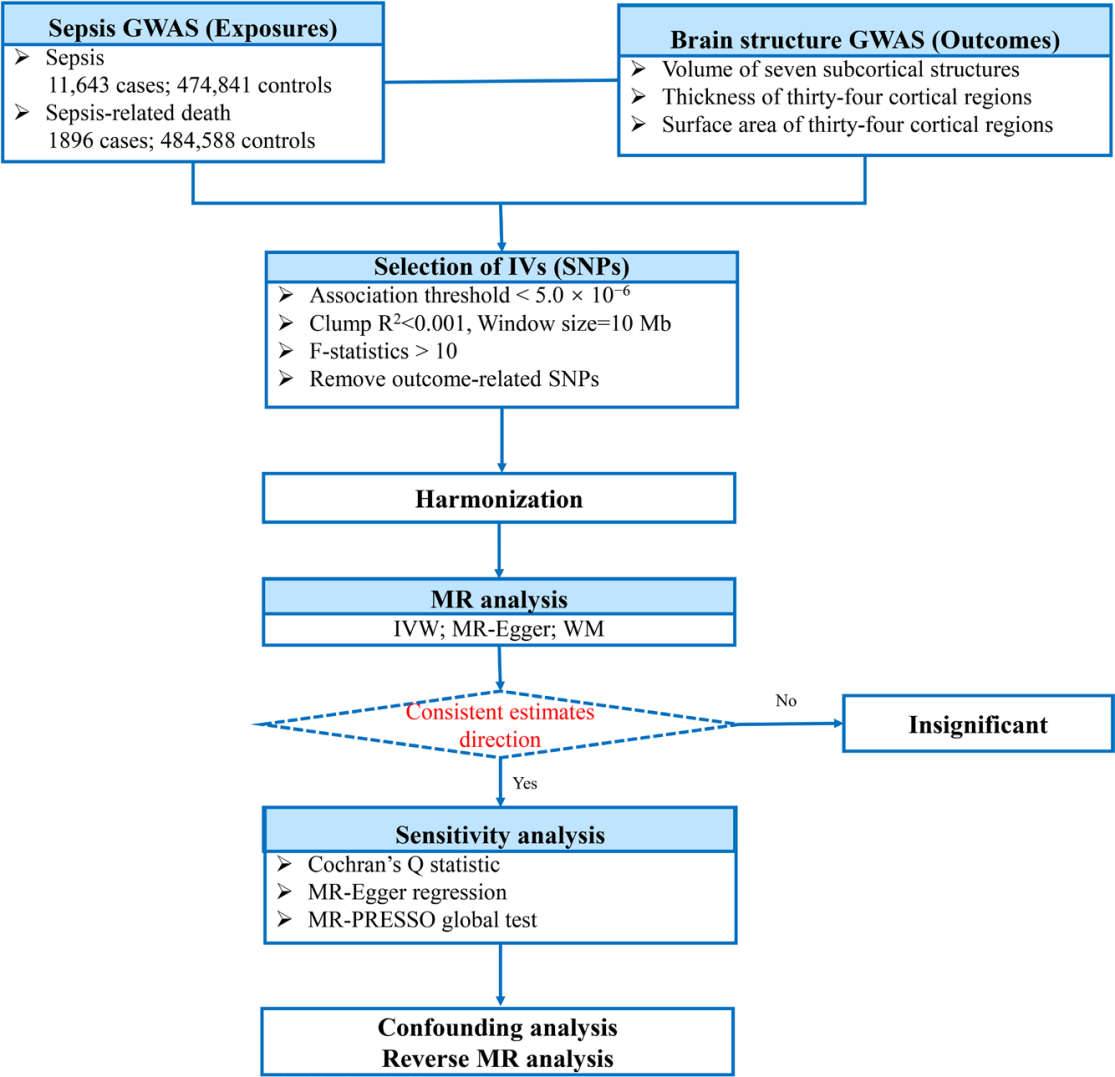
Overall design of our research.

This study followed the guidelines of the Strengthening the Reporting of Observational Studies in Epidemiology Using MR (STROBE-MR), a checklist of which can be found in the Supporting Information (Table S1, Supplemental Digital Content, http://links.lww.com/MD/N414).^[17]^ Employing summary statistics derived from genome-wide association studies (GWAS), we conducted 2-sample MR analyses to evaluate the causality of sepsis, sepsis (28-day death), and brain cortical structure. IVs are chosen to meet 3 assumptions: IVs are required to be strongly associated with the exposure of interest; IVs must be independent of unmeasured confounders; and IVs influence outcomes only through the exposure of interest.^[18]^ In this MR study, sepsis, sepsis (28-day death), and brain cortical structure, and were used as exposure and outcome, respectively. A flowchart depicting the overall design is presented in Figure 1.

### 2.2. Sepsis samples

Summary statistics for sepsis phenotypes were extracted from the IEU OpenGWAS, while summary-level data from the UK Biobank was also utilized (https://gwas.mrcieu.ac.uk/). The UK Biobank is an extensive cohort of UK adult participants, details were found in other sections.^[19]^ Sepsis phenotypes included sepsis (total cases: 11,643; total controls: 474,841) and sepsis-related 28-day mortality (total cases: 1896; total controls: 484,588). In the hospital episode statistics sourced from the UK Biobank, cases were identified when the relevant code was listed as the primary or secondary diagnosis. The analysis was adjusted for age, gender, microchip, and first 10 principal components using regenie v2.2.4.^[20]^ Sepsis admissions identified by ICD codes from the UK Biobank linking secondary care data. In line with existing literature, ICD-10 codes A02, A39, A40, and A41 were used to identify sepsis.^[21]^ The participants of the studies were all of European ancestry.

### 2.3. Brain cortical structure samples

Brain structural characterization data were selected from the GWAS results of MRI-derived brain morphometry performed by the ENIGMA consortium. Regarding cortical thickness and surface area, 51,665 individuals from 60 cohorts worldwide were included. Thirty-four brain regions and the entire cortex were defined using the Desikan–Killiany Cortical Atlas, and estimates were weighted for the entire brain. Regarding the volume of subcortical structures, 7 brain regions were measured for 30,717 participants. All data were adjusted for intracranial volume. Phenotype was defined as the mean estimate of the left and right hemispheres (thickness in millimeters, surface area in square millimeters, and volume of subcortical structures in cubic centimeters).^[22,23]^

### 2.4. Selection of IVs

As the eligible number of IVs was extremely small (*P* < 5 × 10^−8^), a relatively high threshold (*P* < 5 × 10^−6^) was used, which was in line with Zhou et al.^[24]^ In parallel, we performed quality control according to the following steps: First, the linkage disequilibrium threshold was set as 0.001, and the clumping window was 10 Mb. Second, we computed the *F*-statistic to quantify the genetic variation strength and then abandoned single nucleotide polymorphisms (SNPs) with an *F*-statistic of <10, indicating insufficient strength. Third, harmonizing processes were conducted to exclude ambiguous and palindromic SNPs. Ultimately, the brain cortical structure associated with the outcome exhibited a significance level of *P* < 5.0 × 10^−6^, and brain cortical structures containing fewer than 3 SNPs were excluded from the analysis. Subsequently, the remaining SNPs were employed as IVs following the aforementioned steps.

### 2.5. Primary analysis

For the primary analysis, we employed random effects inverse variance weighted (IVW) estimation, which combines the Wald ratio of each SNP to the outcome, assuming all genetic variations are valid. This approach offers the highest power for MR estimation, yet it is susceptible to multidirectional bias.^[25]^ Hence, IVW was used as the primary method to estimate the causal effect of sepsis, sepsis-related 28-day death on structural changes in specific brain regions.

### 2.6. Sensitivity analysis

Sensitivity analyses were subsequently performed to assess the bias of the MR assumptions for the identified significant estimates (*P*_IVW_ < .05). Some other MR analyses, such as weighted median and MR-Egger regression, were also used as complementary methods. MR-Egger regressions can test for multiplicity and considerable heterogeneity of imbalances, whereas for the same change in underexposure, larger sample sizes are required.^[26]^ In situations where at least half of the weighted variance provided by the horizontal pleated product effect is valid, the weighted median method offers consistent estimates of the effect.^[27]^ In addition, sensitivity analyses are also critical in MR studies to evaluate any bias of the MR assumptions. Consequently, Cochran *Q* statistic, and MR-Egger intercept tests are employed to identify the presence of heterogeneity and pleiotropy, as well as evaluate the robustness of the obtained results. Mendelian randomization pleiotropy residual sum and outlier (MR-PRESSO) tests were conducted to test for outliers with potential horizontal pleiotropy. Should any outliers be discovered, they are eliminated to produce unbiased causal estimates from the outlier-corrected MR analysis.^[28]^ Hence, the underlying brain structure impacted by sepsis was recognized as follows: *P*_IVW_＜.05; the direction and amplitude of the 3 MR methods were consistent; no pleiotropy was observed. *P*-values <.05 were considered nominally significant, whereas high-confidence results were those that survived adjustment for multiple testing (Bonferroni correction threshold of 0.05/75). All analyses were run by using the R package TwoSampleMR (version 0.5.6) in R (version 4.2.3).

### 2.7. Confounding analysis and reverse MR analysis

Despite employing various statistical approaches in sensitivity analyses to investigate potential violations of MR assumptions, we also utilized the Phenoscanner V2 website (http://www.phenoscanner.medchsl.cam.ac.uk/) to examine whether sepsis-related SNPs were concurrently associated with multiple common risk factors that might influence MR estimates, including all neurological and psychiatric disorders, hypoxemia, obesity, intelligence, and other potential confounders.^[29,30]^ If the correlation between SNPs and these potential confounders reached a threshold value of *P* < 5 × 10^−6^, IVW was reiterated following the removal of these SNPs to validate the robustness of the findings. Additionally, reverse MR analysis was performed on the sepsis which was found to be causally associated with brain cortical structure in forward MR analysis. The procedure for reverse MR analysis is the same as the MR analysis above.

## 3. Results

After a series of quality control steps, the characteristics of selected SNPs were shown in Table S2, Supplemental Digital Content, http://links.lww.com/MD/N414. *F* statistics of all IVs were more than 10, demonstrating the absence of weak instrument bias.

### 3.1. Causal effect of genetic liability to sepsis on brain structure

**Figure 2.**
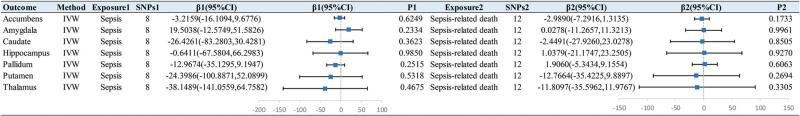
IVW estimates of the effect of sepsis and sepsis-related death on the volume of subcortical structures.

**Figure 3.**
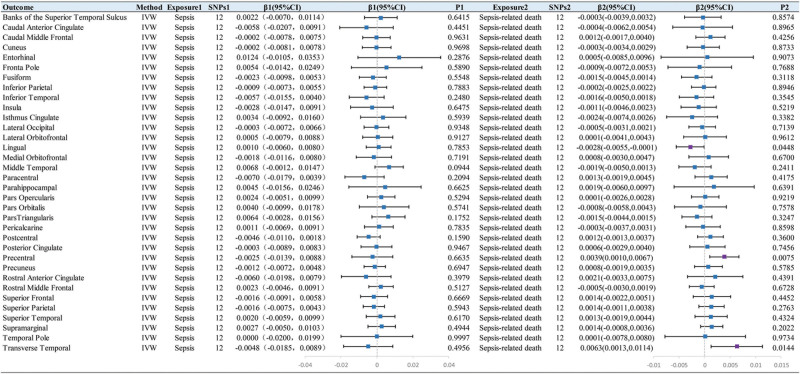
IVW estimates of the effect of sepsis and sepsis-related death on cortical thickness.

**Figure 4.**
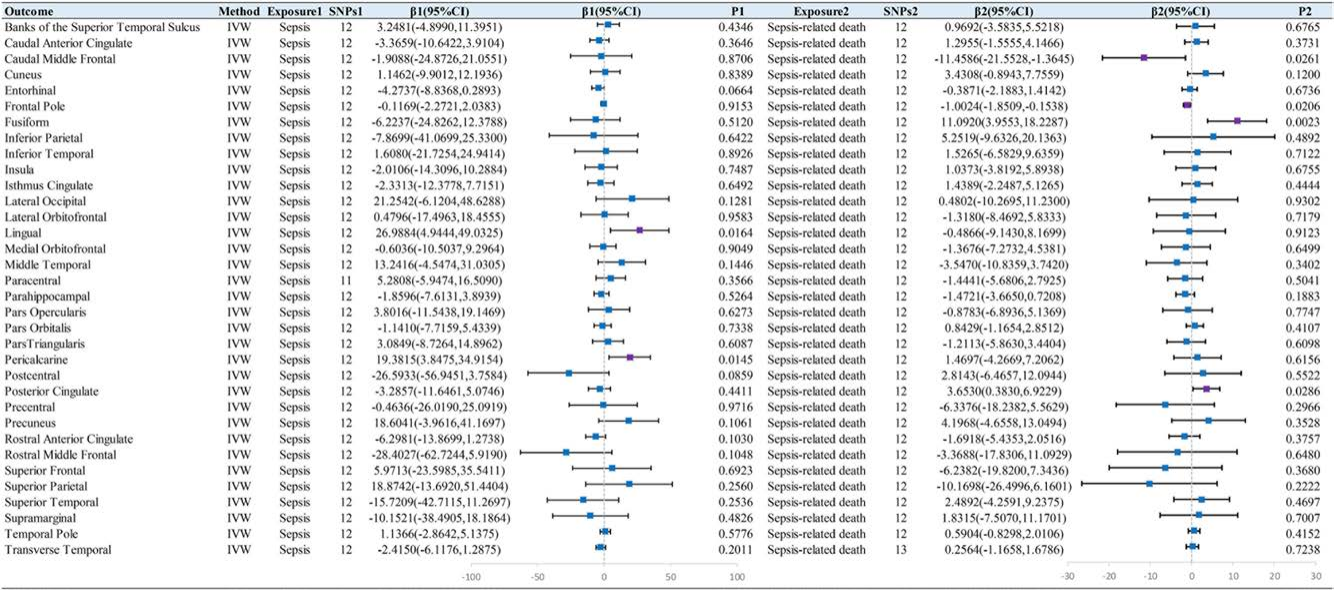
IVW estimates of the effect of sepsis and sepsis-related death on surface area.

Our preliminary IVW analysis showed that genetic liability to sepsis had no causal effect on subcortical structures’ volume or cortical thickness (Figs. 2 and 3), but was causally associated with increased surface area of the lingual gyrus (β = 26.9884, SE = 11.2470, *P* = .0164) and pericalcarine (β = 19.3815, SE = 7.9255, *P* = .0145) (Fig. 4). By performing sensitivity analysis, 3 MR analysis methods identified inconsistent directions of the above effect (Tables S3–S5, Supplemental Digital Content, http://links.lww.com/MD/N414). Interestingly, IVW demonstrated genetic liability to sepsis was associated with reduced surface area of the postcentral gyrus (β = −35.5280, SE = 13.7465, *P* = .0096) after removing SNP outliers identified by the MR-PRESSO method. Cochran *Q*-derived *P* values (*P* > .05) suggested that there was no heterogeneity. Besides, neither the MR-Egger regression test nor the MR-PRESSO test exhibited any evidence of horizontal pleiotropy (all *P* > .05).

### 3.2. Causal effect of genetic liability to sepsis-related death on brain structure

The results of IVW analysis demonstrated that there was no causal relationship between genetic liability to sepsis-related death and subcortical structures (Fig. 2). For cortical thickness, the genetic liability to sepsis-related death was associated with increased thickness of the precentral gyrus (β = 0.0039, SE = 0.0015, *P* = .0075) and transverse temporal gyrus (β = 0.0063, SE = 0.0026, *P* = .0144) as well as reduced thickness of lingual gyrus (β = −0.0028, SE = 0.0014, *P* = .0448) (Fig. 3). However, 3 MR analysis methods showed inconsistency in the direction of the magnitude of the effect of sepsis-related death in relation to the thickness of the precentral gyrus and lingual gyrus (Table S6, Supplemental Digital Content, http://links.lww.com/MD/N414). For cortical surface area, the genetic liability to sepsis-related death exhibited positive correlations with surface area of the fusiform gyrus (β = 11.0920, SE = 3.6412, *P* = .0023) and posterior cingulate gyrus (β = 3.6530, SE = 1.6684, *P* = .0286), While it exhibited negative correlations with surface area of the caudal middle frontal gyrus (β = −11.4586, SE = 5.1501, *P* = .0261) and frontal pole (β = −1.0024, SE = 0.4329, *P* = .0206) (Fig. 4). After Bonferroni correction, all of these results were nominally significant. Moreover, we conducted several sensitivity analyses, including Cochran *Q* test, MR-Egger regression, and MR-PRESSO global test, confirming the rigidity of the above results (Tables S3, S6, and S7, Supplemental Digital Content, http://links.lww.com/MD/N414).

### 3.3. Confounding analysis and Reverse MR analysis

**Figure 5.**
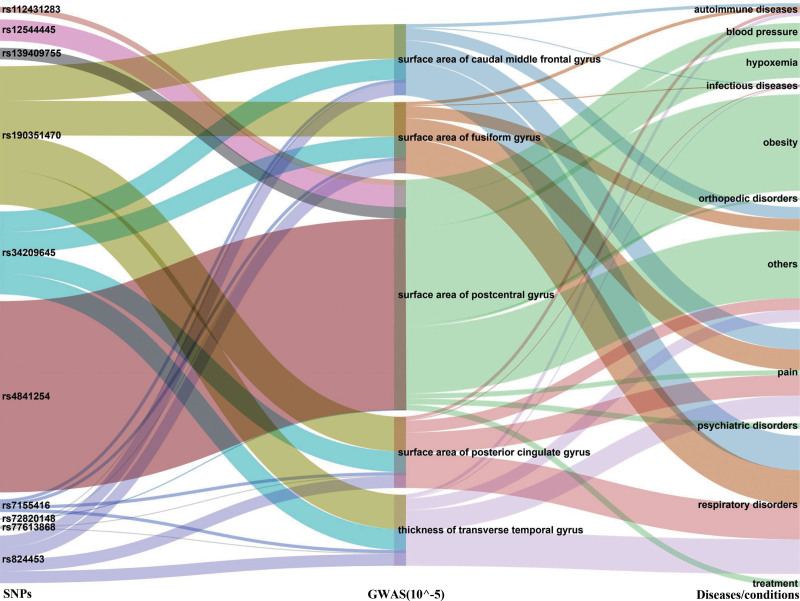
The result of confounding analysis.

Regarding postcentral cortex surface area, 1 SNP (rs4841254) in sepsis phenotype was associated with irritability-related phenotypes. Causality remained significant after removal of this SNP (β_IVW_ = −34.0216, *P* = .02631). Regarding cortex surface area, SNPs of the sepsis-related death phenotype were discovered to be causally related to caudal middle frontal gyrus, frontal pole, fusiform gyrus, and posterior cingulate gyrus cortex surface area, independent of any confounding factors (Fig. 5). No confounders were also found for SNPs related to a causal relationship between genetic liability to sepsis-related death and transverse temporal gyrus cortical thickness. In addition, reverse MR showed a positive correlation between the thickness of the transverse temporal gyrus (β = 3.1983, SE = 1.4157, *P* = .0239) and the risk of sepsis-related death (Table S8, Supplemental Digital Content, http://links.lww.com/MD/N414).

## 4. Discussion

To our knowledge, this is the first study using MR analysis to examine the causal effect of genetic liability to sepsis and sepsis-related death on specific brain region structural changes. Our results showed suggestive associations between genetic liability to sepsis and the postcentral gyrus surface area and sepsis-related death with regional cortical surface area (caudal middle frontal gyrus, frontal pole, fusiform gyrus, and posterior cingulate gyrus). We also found a possible bidirectional causal association between genetic liability to sepsis-related death and the thickness of the transverse temporal gyrus.

For cortical thickness, our study showed no evident associations between genetic liability to sepsis and cortical thickness, but a bidirectional causal association between genetic liability to sepsis-related death and thickness of transverse temporal gyrus. The transverse temporal gyrus contains the primary auditory cortex, which can be damaged by infection, trauma, ischemia, and tumors.^[31,32]^ A cohort study by Kamkwalala et al showed elevated plasma soluble CD14 was associated with volume reductions in several regions of the temporal lobe in women with HIV.^[33]^ This is inconsistent with our findings, suggesting that structural changes in the brain may be caused by pathological processes subsequent to sepsis, such as systemic inflammation, in addition to infection. Furthermore, brain anatomy is closely related to the brain’s ability to resist external pathogens, which may explain the poor prognosis of sepsis due to the increased cortical thickness of transverse temporal gyrus.^[34]^ However, due to a paucity of studies explicitly linking sepsis to the transverse temporal gyrus, we are unable to corroborate the causal relationship discovered.

For cortical surface area, genetic liability to sepsis was suggested to be negatively correlated with surface area of the postcentral gyrus. Whereas the genetic liability to sepsis-related death was normally significantly negatively correlated with surface area of the caudal middle frontal gyrus and frontal pole, and normally significantly positively correlated with surface area of the fusiform gyrus and posterior cingulate gyrus. The postcentral gyrus is the location of the main somatosensory cortex, a significant brain region in charge of proprioception. We hypothesize that sensory dysfunction in patients with sepsis may be mediated by affecting the structure and function of the posterior central gyrus.^[35]^ The caudal middle frontal gyrus and the frontal pole are both part of the frontal cortex. An LPS-induced sepsis rats model by Semmler et al found a reduced density of neurons in the prefrontal cortex during acute sepsis.^[36]^ Additionally, sepsis typically affects prefrontal cortex-related cognitive abilities such as verbal fluency and memory.^[37]^ The posterior cingulate gyrus is a component of the cingulate gyrus, which has been demonstrated to have considerable neuronal loss in SIBD patients.^[38]^ As a crucial node in the salience network of the brain, cingulate gyrus failure can result in apathy and impaired consciousness that are common in sepsis.^[38]^ And there has been little investigation into sepsis and the fusiform gyrus. Moreover, an interesting finding is that these brain structures have been demonstrated in previous studies to be related with Alzheimer disease. A MR study suggested that sepsis significantly increased the risk of Alzheimer disease.^[39]^ We hypothesize that sepsis may influence the development of Alzheimer disease with mediators of specific brain structures.^[40]^ In sum, research related to sepsis and these brain structures is lacking, but there is some indirect evidence of their relevance. Therefore, further research on the structural and functional alterations, as well as related mechanisms of the specific brain structures during sepsis is required.

For subcortical structures, neither genetic liability to sepsis-related death nor the sepsis-related death had a causal relationship with subcortical structure volume. However, contrary to our findings, most previous studies reported reduced volume of subcortical structures such as amygdala, caudate, hippocampus, putamen, and thalamus in sepsis.^[8,38,41]^ The inconsistencies could be attributed to the insufficient sample size as well as the bias of confounding factors and reverse causal associations that are difficult to avoid in observational studies. Based on large sample size GWAS data and minimal confounding factor bias, our study can offer strong support for the inference of causation.

There are several possible mechanisms that might explain how sepsis affects brain structure. Firstly, sepsis may trigger microglia activation via various mechanisms, and notably, the sustained activation of these cells creates a cytotoxic environment, causing neuronal dysfunction and cell death.^[42]^ Secondly, damage to the blood–brain barrier involving intercellular junctions of the endothelial cells may allow peripheral toxic substances to enter the central nervous system, leading to additional damage.^[43]^ Thirdly, microcirculatory dysfunctions in sepsis could lead to insufficient perfusion, resulting in cerebral ischemic necrosis. Post‐mortem study of patients who died of septic shock frequently reveal cerebral ischemic lesions.^[6,44]^ Lastly, the prevalent gut microbiota dysbiosis in sepsis might lead to brain injury via the microbiota-gut-brain axis.^[45]^

The principal strength of our study lies in its utilization of comprehensive MR analysis to elucidate the causal relationship between sepsis and brain structures, effectively circumventing the confounding bias and reverse causal associations inherent in traditional observational studies. Nevertheless, some limitations needed to be considered in the current study. Firstly, due to our reliance on summary-level statistics instead of individual-level data, the study was unable to conduct subgroup analyses. Secondly, the predominance of participants with European ancestry limits the generalizability of our findings to other ethnic groups. Finally, while our study proposes a causal link between sepsis and specific brain structures, it underscores the need for further research to explore potential functional alterations and the mechanisms underpinning these changes.

In summary, our study suggested a potential causal link between genetic liability to sepsis and cortical/subcortical structural changes, adding new evidence for a relationship between sepsis and brain structure changes.

## Acknowledgments

We thank all the authors and participants of GWASs, the online scientific mapping site RAWGraphs 2.0 beta (https://app.rawgraphs.io).

## Author contributions

**Conceptualization:** Dengfeng Zhou, Weina Wang, Qiaofa Lu.

**Data curation:** Dengfeng Zhou, Weina Wang.

**Formal analysis:** Dengfeng Zhou, Weina Wang.

**Investigation:** Dengfeng Zhou, Jiaying Gu.

**Methodology:** Dengfeng Zhou.

**Validation:** Weina Wang.

**Visualization:** Weina Wang.

**Writing – original draft:** Dengfeng Zhou, Weina Wang.

**Writing – review & editing:** Qiaofa Lu.

## Supplementary Material


